# Cocoon Syndrome as a Cause of Intestinal Failure and Indication for Combined Liver–Intestine–Kidney Transplantation

**DOI:** 10.1016/j.intf.2025.100310

**Published:** 2025-10-10

**Authors:** MV Gentilini, M. Doeyo, Santillán Marco, P LC Illidge, F. Pattin, A. Fraile, D. Ramisch, M. Rumbo, H. Solar, GE Gondolesi

**Affiliations:** a Instituto de Medicina Traslacional, Trasplante y Bioingeniería (IMeTTYB), CONICET, Universidad Favaloro, Argentina; b Unidad de Soporte Nutricional, Rehabilitación y Trasplante de intestino, Hospital Universitario Fundación Favaloro, Buenos Aires, Argentina; c Instituto de estudios inmunológicos y fisiopatológicos, IIFP - CONICET, Universidad Nacional de La Plata, Buenos Aires, Argentina; d Pediatric Liver Transplantation and Intestinal Transplantation, MedStar Georgetown University Hospital, Washinton D.C., USA

**Keywords:** Cocoon syndrome, Intestinal transplant, Combined liver-intestinal transplant, Kidney transplant

## Abstract

**Background:**

Cocoon Syndrome, or sclerosing encapsulating peritonitis, is a rare but severe complication of long-term peritoneal dialysis. It can lead to progressive fibrotic encapsulation of the intestines, causing intestinal obstruction due to a fibrous membrane encasing the abdominal organs.

**Case report:**

We present a complex case of a 29-year-old male with history of nephronophthisis and four years on peritoneal dialysis who developed Cocoon Syndrome, leading to intestinal perforation and total enterectomy, resulting in irreversible intestinal failure. After prolonged dependence on parenteral nutrition, he developed intestinal failure-associated liver disease, ultimately becoming a candidate for combined liver–intestine and kidney transplantation.

**Conclusion:**

This case illustrates how advanced Cocoon Syndrome can lead to severe and progressive complications that may ultimately require combined liver–intestine and kidney transplantation. It underscores the importance of early recognition and appropriate multidisciplinary management to improve patient outcomes.

## Introduction

The first report of sclerosing encapsulating peritonitis (SEP) was published in 1921 by Winnen [1]; he described it as “the icing gut” due to intestinal surface appearing white for the membrane covering. In 1978, Foo et al. named “abdominal cocoon” to describe encapsulation of the abdominal contents [2]. SEP is an acquired inflammatory peritoneal condition of unknown etiology believed to result from recurrent low-grade or subclinical peritonitis with no specific abdominal signs that eventually progresses to sclerosis and membrane formation with subsequent cocoon formation. It is characterized by a fibro-collagenous membrane that involves abdominal viscera, and it has been reported as a rare and severe cause of intestinal obstruction [3], [4], [5], [6], [7], [8]. It has been classified as: 1) idiopathic or primary SEP, often referred as abdominal cocoon syndrome. It has an embryonic origin with abnormal mesoderm differentiation and intestinal dorsal mesenteric dysplasia. This condition is associated with other anatomical abnormalities such as the absence of omentum, gastrocolic ligament, visceral transposition, intestinal and colonic malrotation; 2) secondary SEP when there are several risk factors as tuberculosis, neoplasm, beta blockers use, peritoneal dialysis (PD) and previous abdominal surgeries that cause peritoneum inflammation, neo angiogenesis and fibroblastic proliferation secondary to cytokine release and fibroblast activation resulting in the formation of a thick fibrocollagenous peritoneal membrane [9], [10].

There are three types of abdominal cocoon: type I is when partial encapsulation of the intestine occurs; type II when complete encapsulation of the entire intestine occurs and type III when encasement of the entire intestine and other intra-abdominal organs such as appendix, ascending colon, stomach, liver and ovaries occur.

In the early stages the diagnosis is difficult as symptoms are non-specific, however in more advanced stages recurrent episodes of abdominal pain and intestinal obstruction due to kinking or compression of the bowel caused by the fibro-collagenous membrane are present, in some cases the membrane can be calcified. The degree of intestinal involvement may be severe and progress to ischemia and perforation, increasing the morbidity-mortality. In many cases, preoperative diagnosis is difficult, and the diagnosis is performed during exploratory laparotomy.

Management of abdominal cocoon syndrome is not well established. A conservative treatment has been proposed for patients with mild symptoms (nasogastric tube decompression, bowel rest and hydration); when symptoms are severe adhesiolysis and excision of the covering membrane, to free the entire bowel is indicated, not always can be achieved. In other cases, due to the presence of bowel ischemia or perforation, the resection of the intestine could be required [11].

Peritoneal dialysis (PD) is a known risk factor for secondary sclerosing encapsulating peritonitis (SEP), particularly with long-term use. Chronic exposure to bioincompatible dialysates, recurrent peritonitis, and sustained peritoneal inflammation can lead to progressive fibrosis and membrane calcification. The incidence of SEP increases with PD duration—from around 2 % after two years to nearly 20 % after eight years—highlighting the cumulative risk associated with prolonged therapy.

In 1990 the mortality of SEP was reported nearly to 60 % when is related to surgical complications such as intestinal obstruction, intestinal necrosis and enterocutaneous fistulas [12]. However, it did not improve through the years and despite various therapeutic modalities and mortality continue been between 56 % and 93 % [13].

The intestinal resection can lead patients to have short bowel syndrome (SBS) and intestinal failure (IF) dependent on home parenteral nutrition (HPN). If patients develop complications associated with this support, intestinal or multivisceral transplants become the last alternative therapy.

We aim here to present a case of cocoon syndrome in a young patient with a history of nephronophthisis, chronic kidney failure and chronic PD who developed a type II IF, which progressed to type III as result of the extensive calcifications. Consequently, intestinal obstruction, intestinal ischemia, perforation and peritonitis occur requiring an extense enterectomy evolving to SBS, chronic intestinal failure (CIF) depending on HPN, and ultimately developed IFALD. He was subsequently listed and transplanted with a combined liver–intestine and kidney graft.

## Case report

A 29-year-old male with a history of chronic kidney disease secondary to nephronophthisis, diagnosed in 1996, and essential hypogammaglobulinemia, had been on peritoneal dialysis (PD) since 2006. He was initially referred to our center for evaluation as a candidate for kidney transplantation.

In 2010, after four years on PD, the patient presented with symptoms of intestinal subocclusion. An abdominal CT scan was performed, and SEP type I was diagnosed Conservative management was implemented, including nasogastric tube decompression, intestinal rest, and intravenous hydration. PD was discontinued and replaced with hemodialysis.

**Fig. 1:**
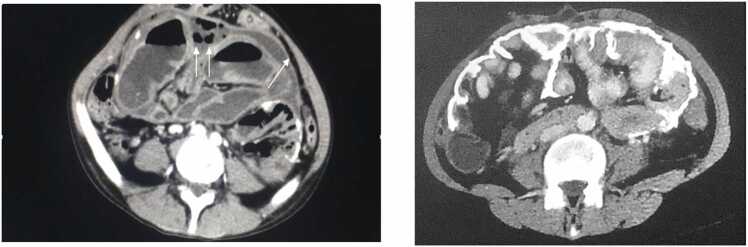
Computed tomography (CT) axial views. On the left, the CT image corresponds to the patient described in the present case, showing extensive peritoneal thickening and signs of peritoneal encapsulation consistent with early sclerosing encapsulating peritonitis (SEP). On the right, an illustrative image from a different patient is shown to depict the typical CT findings associated with advanced-stage SEP, including marked peritoneal calcifications, encapsulated bowel loops, and a cocoon-like appearance. This comparison highlights the spectrum of radiological manifestations of SEP.

In April 2014, the patient underwent parathyroidectomy due to secondary hyperparathyroidism. Later that year, in November 2014, he was readmitted with acute abdominal occlusion that did not resolve with medical management requiring an exploratory laparotomy. During surgery an extensive calcification of the cocoon membrane with encapsulation of all the small bowel (SEP Type III) was identified as soon as the abdomen was open, an intestinal ischemia with a perforation and secondary peritonitis were the final finding (Fig. 1). An extense enterectomy was required, leaving a remnant of only 10 cm of small bowel to terminal jejunostomy (anatomy type I), and a preserved only a segment of sigmoid colon. Thus, the patient became dependent on parenteral nutrition (PN).

In 2015, he was evaluated for organ transplantation and initially listed for isolated intestinal and kidney transplantation. The patient subsequently developed intestinal failure-associated liver disease (IFALD), confirmed by biopsy (Metavir F3), prompting to change his listing criteria for a combined liver–intestine–kidney transplantation.

After 1125 days on the waiting list, he underwent in bloc transplantation from an 11-year-old ABO-compatible donor.

## Discussion

Primary SEP is a rare clinical condition and there are approximately 200 reported cases in the English literature [4]. Secondary SEP is a more common clinical condition and is associated with several causes; however, the predominant cause is PD. Long PD duration, acetate-buffered or hypertonic solutions and recurrent episodes of clinical and subclinical peritonitis are known contributors and may predispose patients to develop SEP.

In this setting, the risk of abdominal complications increases, and effectivity of ultrafiltration decrease due to the presence of an encasing membrane [14]. Those membranes lead to a progressive development of adhesions, up to the point where the intestine becomes encapsulated, and like in our case, and probably in association to the hypercalcemia secondary to his hyperparathyroidism, the peritoneal membrane become calcified.

Our patient developed symptoms of intestinal obstuction after four years on PD, at which time abdominal CT imaging revealed a fibrous membrane involving the abdominal viscera, consistent with SEP type II. PD was discontinued and replaced by hemodialysis. Over the following four years, despite cessation of PD, the disease progressed insidiously, with increasing peritoneal calcifications. Eight years after the initiation of PD, he was presented with acute intestinal obstruction. At laparotomy, the SEP had evolved to type III, involving the appendix and right colon, and extensive intestinal resection was required.

Despite surviving most of these complications, the patient ultimately developed two benign but irreversible conditions: chronic kidney failure requiring hemodialysis three times per week, and CIF secondary to SBS, necessitating lifelong HPN for 16 h daily, *without the possibility of reconstructing his intestinal transit*.

To the best of our knowledge there are a few reports on SEP, SBS and CIF. In 1990 Dillpi S. Kittur reported a patient with SEP who required extensive small intestinal resection (more than 240 cm), but the patient died [15]. Similarly, in a publication of 2007, from three surgical cases, the patient who required intestinal resection due to intestinal perforation was who had the worst evolution dying one month later of the last surgery [16]. In both reports, wide intestinal resections with primary anastomosis were performed. Failure of these anastomoses with leakage resulted in secondary peritonitis, septic shock and death. Other surgical reports did not perform intestinal resection but rather bowel release or membrane resection.

On the other hand, only one case has been published describing a patient with SEP secondary to PD who underwent combined intestinal and kidney transplantation due to progressive intestinal failure [17]. Notably, that patient did not undergo enterectomy and did not develop liver dysfunction. In contrast, the present case is, to our knowledge, the first to describe a patient with type III SEP, complicated by intestinal perforation and total enterectomy, who survived chronic intestinal failure and hemodialysis dependence. The subsequent development of intestinal failure–associated liver disease (IFALD), confirmed by liver biopsy (Metavir F3), was likely driven by a multifactorial burden—including persistent systemic inflammation, recurrent infections, severe malnutrition, and long-term parenteral nutrition—despite adherence to optimized nutritional management. This clinical trajectory ultimately led to the indication for combined liver, intestine, and kidney transplantation.

This report underscores several critical clinical insights. First, it emphasizes the need for vigilance in patients on long-term PD, particularly those with recurrent peritonitis, due to the risk of developing SEP. Although rare, intestinal perforation and catastrophic intestinal loss can occur even without classical signs of acute peritonitis, especially in patients with visible peritoneal calcifications. Surgical management in these scenarios typically involves dissection of the fibrotic membrane and adhesiolysis, with resection reserved for non-viable bowel segments due to its inherent morbidity and mortality. In this case, advanced disease with diffuse ischemic injury precluded any conservative strategy, necessitating total enterectomy and colectomy despite the presence of a single perforation.

In conclusion, this case underscores the importance of early recognition of SEP in patients on long-term PD, especially those with recurrent peritonitis or peritoneal calcifications. Although uncommon, progression to irreversible intestinal damage, chronic intestinal failure, and IFALD may occur, ultimately requiring combined liver–intestine–kidney transplantation Early diagnosis and timely multidisciplinary management remain essential to prevent progression and improve outcomes.

## CRediT authorship contribution statement

**Gentilini Maria Virginia:** Writing – review & editing, Writing – original draft, Visualization, Validation, Supervision, Software, Resources, Methodology, Investigation, Formal analysis, Data curation, Conceptualization. **Doeyo Mariana:** Writing – original draft, Methodology, Investigation, Data curation. **Santillán Marco:** Methodology, Investigation. **Pattin Francisco:** Methodology, Investigation. **Fraile Andrés:** Methodology, Investigation. **Ramisch Diego:** Methodology, Investigation. **Rumbo Martín:** Methodology, Investigation. **Solar Héctor:** Methodology, Investigation, Conceptualization. **GE Gondolesi:** Writing – review & editing, Writing – original draft, Visualization, Validation, Supervision, Software, Resources, Project administration, Methodology, Investigation, Funding acquisition, Formal analysis, Data curation, Conceptualization. **P LC Illidge:** Methodology and Investigation.

## Patient's consent

Written informed consent was obtained from the individual. The study was approved by our Institutional Review Board (approval number 1477–1119).

## Ethical statement

The study was approved by the Ethics Committee of our Institutional Review Board (approval number 1477–1119).

The studies were conducted in accordance with the local legislation and institutional requirements. Written informed consent for participation was required from the participant in accordance with the national legislation and institutional requirements.

## Declaration of Generative AI and AI-assisted technologies in the writing process

During the preparation of this work the authors used ChatGPT to improve the language. After using this tool/service, the author(s) reviewed and edited the content as needed and take full responsibility for the content of the publication.

## Funding

This research did not receive any specific grant from funding agencies in the public, commercial or non-profit sectors.

## Declaration of Competing Interest

The authors declare that they have no known competing financial interests or personal relationships that could have appeared to influence the work reported in this paper.

## References

[1] Winnen P. Uber Zuckergussdarm (peritonitis chronica fibrosa incapsulata). Brunns Beitr Klin Chir 1921; 123:72).

[2] Foo K.T., Ng K.C., Rauff A., Foong W.C., Sinniah R. (1978). Unusual small intestinal obstuction in adolescent girls: the abdominal cocoon. Br J Surg.

[3] Tannoury J.N., Abboud B.N. (2012). Idiopathic sclerosing encapsulating peritonitis: abdominal cocoon. World J Gastroenterol.

[4] Akbulut S. (2015). Accurate definition and management of idiopathic sclerosing encapsulating peritonitis. World J Gastroenterol.

[5] Kawanishi H., Watanabe H., Moriishi M., Tsuchiya S. (2005). Successful surgical management of encapsulating peritoneal sclerosis. Perit Dial Int.

[6] Oran E., Seyit H., Besleyici C., Ünsal A., Aliş H. (2015). Encapsulating peritoneal sclerosis as a late complication of peritoneal dialysis. Ann Med Surg.

[7] Li N. (2014). Surgical treatment and perioperative management of idiopathic abdominal cocoon: Single-center review of 65 cases. World J Surg.

[8] Maguire D., Srinivasan P., O’Grady J., Rela M., Heaton N.D. (2001). Sclerosing encapsulating peritonitis after orthotopic liver transplantation. Am J Surg.

[9] Vagholkar K., Doctor-Ganju K. (2018). Abdominal cocoon: a surgical challenge. Int Surg J.

[10] Frazão J., Martins A.R., Calado J., Godinho A. (2022). Abdominal cocoon syndrome: a rare cause of intestinal obstruction. Cureus.

[11] Sharma D., Nair R.P., Dani T., Shetty P. (2013). Abdominal cocoon - a rare cause of intestinal obstruction. Int J Surg Case Rep.

[12] Kittur D.S., Korpe S.W., Raytch R.E., Smith G.W. (1990). Surgical aspects of sclerosing encapsulating peritonitis. Arch Surg.

[13] Noormohamed M.S., Kadi N. (2012). Abdominal cocoon in peritoneal dialysis - a fatal outcome. BMJ Case Rep.

[14] Kawaguchi Y., Kawanishi H., Mujais S., Topley N., Oreopoulos D.G. (2000). Encapsulating peritoneal sclerosis: definition, etiology, diagnosis, and treatment. International society for peritoneal dialysis ad hoc committee on ultrafiltration management in peritoneal dialysis. Perit Dial Int.

[15] Kittur D.S., Korpe S.W., Raytch R.E., Smith G.W. (1990). Surgical aspects of sclerosing encapsulating peritonitis. Arch Surg.

[16] Bujalance Cabrera F.M. (2007). Tratamiento quirúrgico de la peritonitis esclerosante. Cir Esp.

[17] Waghray A. (2013). Combined intestine and kidney transplantation in a patient with encapsulating peritoneal sclerosis: case report. Am J Transplant.

